# Maximum Somatic Allele Frequency-Adjusted Blood-Based Tumor Mutational Burden Predicts the Efficacy of Immune Checkpoint Inhibitors in Advanced Non-Small Cell Lung Cancer

**DOI:** 10.3390/cancers14225649

**Published:** 2022-11-17

**Authors:** Yiting Dong, Yixiang Zhu, Minglei Zhuo, Xiaomin Chen, Yinpeng Xie, Jianchun Duan, Hua Bai, Shiguang Hao, Zicheng Yu, Yuting Yi, Yanfang Guan, Jie Yuan, Xuefeng Xia, Xin Yi, Jie Wang, Zhijie Wang

**Affiliations:** 1 State Key Laboratory of Molecular Oncology, Department of Medical Oncology, National Cancer Center/National Clinical Research Center for Cancer/Cancer Hospital, Chinese Academy of Medical Sciences & Peking Union Medical College, Beijing 100021, China; 2 Key Laboratory of Carcinogenesis and Translational Research (Ministry of Education/Beijing), Department I of Thoracic Oncology, Peking University Cancer Hospital & Institute, Beijing 100142, China; 3 Geneplus-Shenzhen, Shenzhen 518118, China; 4 Geneplus-Beijing, Beijing 102200, China

**Keywords:** non-small-cell lung cancer, NSCLC, maximum somatic allele frequency, MSAF, tumor mutation burden, TMB, immune checkpoint inhibitors, ICIs

## Abstract

**Simple Summary:**

Recent studies exhibited the unstable prediction ability of blood-based tumor mutational burden (bTMB) when predicting the response of immune checkpoint inhibitors (ICIs) therapy in patients with non-small cell lung cancer (NSCLC). Circulating tumor DNA (ctDNA) abundance, usually represented by maximum somatic allele frequency (MSAF), was one possible confounding factor influencing bTMB ability in ICIs response prediction. We herein developed a novel approach to optimize the calculation of bTMB by integrating MSAF, namely, MSAF-adjusted bTMB (Ma-bTMB), to better select beneficiaries of ICIs. Our present results showed that this novel non-invasive biomarker could reduce the confounding effect of MSAF and ITH on bTMB calculation and effectively identify beneficiaries of ICIs in patients with advanced NSCLC, warranting future clinical trials.

**Abstract:**

Introduction: Recent studies exhibited the unstable prediction ability of blood-based tumor mutational burden (bTMB) when predicting the response of immune checkpoint inhibitors (ICIs) therapy in patients with non-small cell lung cancer (NSCLC). Circulating tumor DNA (ctDNA) abundance, usually represented by maximum somatic allele frequency (MSAF), was one possible confounding factor influencing bTMB ability in ICIs response prediction. Methods: MSAF-adjusted bTMB (Ma-bTMB) was established and validated in patients with advanced NSCLC among Geneplus Cancer Genome Database (GCGD, *n* = 1679), Zhuo (*n* = 35), Wang (*n* = 45), POPLAR (NCT01903993, *n* = 211) and OAK (NCT02008227, *n* = 642) cohorts. Results: MSAF demonstrated a modest positive correlation with bTMB and a negative one with survival benefit. Improved survival outcomes of ICIs therapy have been observed among patients with high-Ma-bTMB compared to those with low-Ma-bTMB in Zhuo and Wang cohorts. In addition, compared to low-Ma-bTMB, high-Ma-bTMB was associated with more positive clinical benefits from ICIs therapy than chemotherapy both in POPLAR and OAK cohorts. Further exploration suggested that Ma-bTMB could precisely identify more potential ICIs beneficiaries compared to bTMB and LAF-bTMB, complementary to PD-L1 expression. Conclusions: We developed Ma-bTMB, a convenient, readily available, non-invasive predictive biomarker effectively differentiates beneficiaries of ICIs therapy in advanced NSCLC, warranting future clinical trials.

## 1. Introduction

Immune checkpoint inhibitors (ICIs), targeting programmed death-ligand 1 (PD-L1) or programmed death 1 (PD-1), have been found to be associated with improved overall survival (OS) of patients with advanced non-small cell lung cancer (NSCLC) [1,2,3]. However, evidence showed that only a few patients could benefit from ICIs treatment, and identifying predictors of favorable ICIs treatment outcomes is of great importance.

Though PD-L1 expression and tissue-based tumor mutational burden (tTMB) have been proven to be useful predictive biomarkers for patients with advanced NSCLC treated with ICIs [4,5], sufficient high-quality tissue samples for standard biomarker analysis were available only for a minority of patients [6]. Hence, developing a non-invasive, convenient, and safe method is urgently needed. Blood-based TMB (bTMB) is of great interest as a candidate in place of tTMB, whereas the results showed that bTMB could not predict OS benefit for ICIs treatment [7,8]. A recently updated result of the phase 2 B-F1RST trial showed that bTMB failed to predict both OS and progression-free survival (PFS) of patients with NSCLC receiving ICIs at a median 36.5-month follow-up [9]. Moreover, Wei Nie et al. found an upside-down U-shaped curve between bTMB and survival, indicating that patients with lower or higher bTMB had longer PFS and OS compared with those with medium bTMB [10]. In short, the information above revealed the unstable prediction ability of bTMB when predicting the survival benefit of ICIs therapy.

Different from tTMB obtained through local tumor biopsy, bTMB is calculated based on circulating tumor DNA (ctDNA) derived from all tumor niches, implying intratumor heterogeneity (ITH) and tumor burden in a patient with advanced cancer [11,12]. ctDNA abundance, which is difficult to directly measure and usually represented by maximum somatic allele frequency (MSAF), has been thought to estimate tumor content, displaying a positive correlation with bTMB while negatively correlating with survival [7,13]. In addition, ITH refers to distinct subclones or subclonal mutations resulting from tumor evolution within the same tumor-bearing patient, showing a correlation with poor prognosis [14,15]. Hence, there is an inner connection between ctDNA abundance (MSAF as an alternative) and ITH as poor prognosis confounding factors in bTMB prediction for ICIs efficacy in patients with advanced NSCLC.

With the development of targeted genomic sequencing assays, a simple, steady, and propagable modified bTMB algorithm that could identify both OS and PFS beneficiaries from ICIs treatment across various panels and calling principles is needed. In this study, we identified MSAF as the core confounding factor of bTMB in survival prediction of ICIs therapy, according to which we established and validated a novel predictive biomarker MSAF-adjusted bTMB (Ma-bTMB) in patients with advanced NSCLC among Zhuo, Wang, POPLAR, and OAK cohorts. Finally, the application of Ma-bTMB was compared with that of other bTMB biomarkers and PD-L1 expression.

## 2. Methods and Materials

### 2.1. Patient Enrollment and Sample Collection

Eighty patients with stage III–IV NSCLC were enrolled from two centers, Peking University Cancer Hospital & Institute (Zhuo cohort, *n* = 35) and Cancer Hospital of Chinese Academy of Medical Sciences & Peking Union Medical College (Wang cohort, *n* = 45) from 25 August 2016 to 12 April 2019. Patients with MSAF = 0 were excluded due to incalculableness (Zhuo cohort, *n* = 4; Wang cohort, *n* = 5). All patients were pathologically diagnosed and underwent anti-PD-(L)-1 therapy. The baseline characteristics, mutational information, and clinical information of Wang and Zhuo cohorts are presented in Tables S1–S3. Peripheral blood samples were collected for 1021-gene-panel genomic analysis before ICI administration. The study was designed and conducted in accordance with the Declaration of Helsinki and approved by the Institutional Review Board (IRB) of Cancer Hospital of Chinese Academy of Medical Sciences & Peking Union Medical College (NCC2018–092) and Peking University Cancer Hospital & Institute (2017KT57). Written informed consent was granted prior to sample collection, genetic sequencing, and data analysis from each patient. 

### 2.2. Datasets and Cohorts

To analyze the association between MSAF and bTMB, somatic single nucleotide variations (SNVs) and indels detected in the ctDNA by 1021-gene-panel from the plasma of 1679 Chinese NSCLC patients enrolled from 1 July 2018 to 12 March 2020, were downloaded from Geneplus Cancer Genome Database (GCGD) cohort (*n* = 1679, accessed in October 2020), which is an internal database of Geneplus Co., Ltd. (Bangkok, Thailand). To verify that Ma-bTMB is associated with ICIs rather than chemotherapy response, POPLAR (NCT01903993) and OAK (NCT02008227) cohorts, two large randomized clinical trials investigating the efficacy and safety of atezolizumab (MPDL3280A, anti-PD-L1) compared with docetaxel (chemotherapy) in patients with NSCLC, were obtained from a published study and included in the subsequent algorithm analysis [7]. Different from the 1021-gene-panel, baseline bTMB in these two public cohorts was sequenced through FDA-approved FoundationOne (F1) CDx NGS assay (POPLAR, N = 211; OAK, *n* = 642). Among these patients, 106 received docetaxel therapy and 105 received atezolizumab therapy in the POPLAR cohort, with 318 vs. 324, respectively, in the OAK cohort [7]. Detailed mutational and clinical information on these two cohorts is presented in Table S4. PD-L1 expression was only available in OAK cohort; PD-L1-positive expression was defined as PD-L1 expression on ≥1% of tumor cells (TCs) or ≥1% of tumor-infiltrating immune cells (ICs).

### 2.3. Targeted Capture Sequencing and Genomic Data Analysis

Blood samples were collected in EDTA vacutainer tubes (BD Diagnostics, Franklin Lakes, NJ, USA) and subjected to ctDNA extraction and sequencing within 3 hours. Peripheral blood lymphocytes and plasma were separated via sequential centrifugation (2500× *g*, 10 min; 16,000× *g*, 10 min). Next, circulating free DNA (cfDNA) was extracted using a DNeasy Blood Kit (Qiagen, Hilden, Germany) and QIAamp Circulating Nucleic Acid Kit (Qiagen). White blood cells from the same patient were used as control. Sequencing libraries were constructed utilizing the KAPA DNA Library Preparation Kit (Kapa Biosystems, Wilmington, MA, USA) per the manufacturer’s instructions. Barcoded libraries were subsequently hybridized to a customized panel of 1021 genes, consisting of whole exons and selected introns of 288 genes, along with selected regions of 733 genes. A detailed gene list was described in our previous study [16]. DNA sequencing was performed with the HiSeq 3000 Sequencing System (Illumina, San Diego, CA, USA) with 2 × 101 bp paired-end reads. For each mutation, somatic allele frequency was calculated by dividing the number of mapped mutational reads by the average sequencing depth. The number of somatic coding non-synonymous SNVs, small insertions, and deletions per megabase (Muts/Mb) of each genome examined was calculated as bTMB, and germline polymorphisms were removed for calculating bTMB. Then, MSAF-adjusted bTMB was calculated as Ma-bTMB = bTMB / (MSAF × 10). The number 10 is a manually selected constant, which could scale down the values of Ma-bTMB to roughly fall within a range similar to other reported bTMB values.

### 2.4. Subclone and Subclonal Mutation Calculation

PyClone was employed to estimate clonal and subclonal populations [17]. We defined a subclone as a collection of cells in the tumor sample that harbored the same set of genomic variants, including SNVs, insertions, and deletions. Subclonal mutations were the total mutated genes of corresponding subclone clusters.

### 2.5. Statistics

Efficacy evaluation was performed by using RECIST version 1.1, and PFS was defined as the time from the start of anti-PD-(L)1 treatment until either objective disease progression (assessed by an investigator using RECIST version 1.1) or death from any cause. OS was defined as the time from the start of anti-PD-(L)1 treatment until death from any cause. We censored patients who had not progressed, who had died at the time of statistical analysis, or who failed to follow up but had not died at the time of their last evaluation.

Pearson correlation tests were employed to determine the association between MSAF and OS/PFS or bTMB. Kaplan–Meier curves were plotted and, together with log-rank tests, performed to calculate the hazard ratios (HRs) and evaluate the prognostic value of various markers. The interaction *p*-value was obtained from an unstratified proportional Cox model, including the terms of treatment, the biomarker subgroup, and treatment by the biomarker subgroup interaction. For all analyses, *p* < 0.05 was considered statistically significant. IBM SPSS software (version 24.0), GraphPad Prism (version 9.0), and R 4.2.0 were used for the statistical analysis.

## 3. Results

### 3.1. Study Design and Cohorts

**Figure 1:**
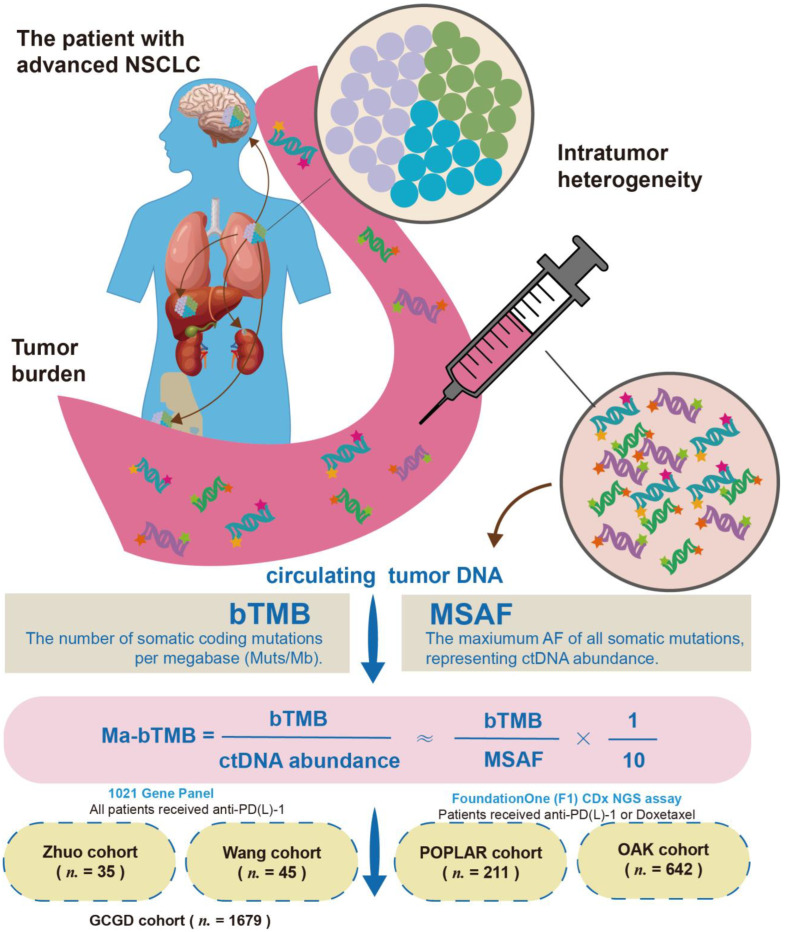
Study design. The patient with advanced NSCLC is characterized by high tumor burden and intratumor heterogeneity (ITH). Circulating free DNA (cfDNA) was obtained for ITH, bTMB, and MSAF analysis. Finally, the algorithm Ma-bTMB was explored and validated in 71 patients receiving anti-PD-(L)-1 from Zhuo and Wang cohorts and 873 patients receiving anti-PD-(L)-1 or chemotherapy from POPLAR and OAK cohorts.

The study design is illustrated in Figure 1. The advanced NSCLC patient is characterized by high tumor burden and ITH. cfDNA was obtained for ITH, bTMB, and MSAF analysis, according to which Ma-bTMB was established. Patients with MSAF = 0 were excluded due to incalculableness. Finally, the algorithm Ma-bTMB was explored and validated in 71 patients with advanced NSCLC receiving anti-PD-(L)-1 from Zhuo (discovery cohort, *n* = 31) and Wang (validation cohort, *n* = 40) cohorts and 851 patients with advanced NSCLC receiving anti-PD-(L)-1 or chemotherapy from POPLAR (discovery cohort, *n* = 209) and OAK (validation cohort, *n* = 642) cohorts. PD-L1 expression was only available in the OAK cohort (*n* = 635).

### 3.2. bTMB, MSAF, and ITH Analysis

**Figure 2:**
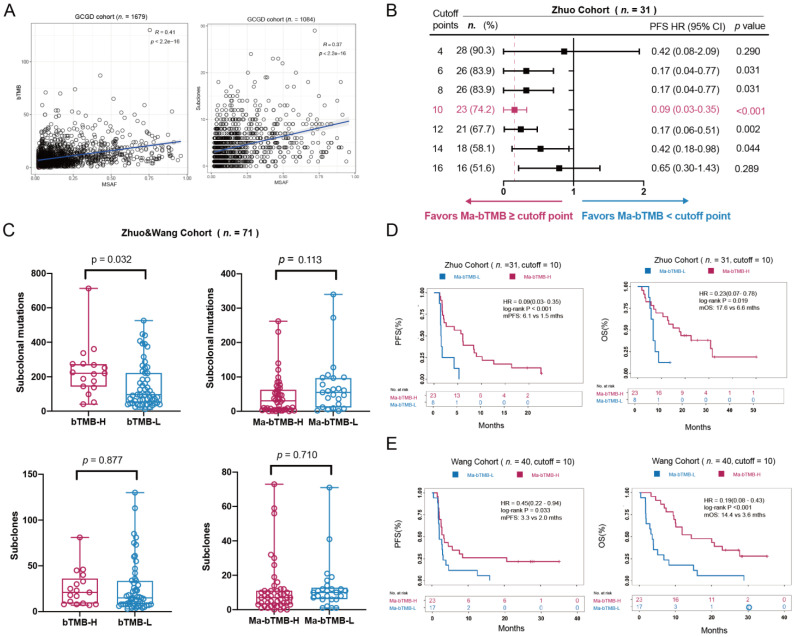
Primary Analysis in Wang and Zhuo cohorts. (**A**) Correlations between MSAF and bTMB /subclones in the GCGD cohort. (**B**) Forest plot for hazard ratios (HRs) and *p*-values of PFS comparing Ma-bTMB-H and Ma-bTMB-L with corresponding cutoff points in Zhuo cohort. (**C**) Distribution of subclones and subclonal mutations of bTMB and Ma-bTMB subgroups in Zhuo & Wang cohort. (**D**) Kaplan–Meier (K–M) curves of PFS and OS between Ma-bTMB-H and Ma-bTMB-L groups in Zhuo cohort. (**E**) K–M curves of PFS and OS between Ma-bTMB-H and Ma-bTMB-L groups in Wang cohort.

In the Zhuo cohort as the discovery cohort, we found that bTMB could not identify either PFS or OS benefits from ICIs (Figure S1); similar results were observed in Wang cohort. Notably, MSAF was found a significant correlation with poor OS (R = −0.42, *p* < 0.001) and PFS (R = −0.28, *p* = 0.017; Figure S2). Moreover, there was a positive correlation between MSAF and bTMB (R = 0.41, *p* < 0.001) as well as between MSAF and subclones (R = 0.37, *p* < 0.001) in GCGD cohort (Figure 2A and Figure S3), indicating that MSAF might be the core confounding factor in the bTMB prediction of ICIs effect. Therefore, we developed the modified biomarker Ma-bTMB by dividing a patient’s bTMB value with the corresponding MSAF and bTMB (Figure 1).

To explore whether Ma-bTMB balances the poor prognosis effect of ITH, we compared subclones and subclonal mutations between high and low groups divided by bTMB and Ma-bTMB. A 25% increase in the bTMB value with a cutoff score of 14 was defined as the cutoff point; the Ma-bTMB cutoff score of 10 was selected as the HR and *p*-value reaching a minimum in PFS prediction compared with other cutoffs (Figure 2B). Even though no significant differences in subclones numbers were observed between high-bTMB and low-bTMB groups (Mean: 25.3 vs. 26.4, *p* = 0.877), there was a higher level of subclonal mutations in the high-bTMB group than in the low-bTMB group (Mean: 234.2 vs. 150.7, *p* = 0.032) in Wang and Zhuo cohorts (hereafter referred to as Wang & Zhuo cohort; Figure 2C). As for Ma-bTMB, no difference was observed between high- and low-Ma-bTMB groups regardless of subclones (Mean: 11.6 vs. 13.0, *p* = 0.710) or subclonal mutation level (Mean: 43.9 vs. 70.1, *p* = 0.113), suggesting that Ma-bTMB could adjust bTMB by balancing the effect of ITH on stratification.

### 3.3. Ma-bTMB as a Prognosis Biomarker for Patients Receiving Anti-PD-(L)1 Therapy

We first investigated the prediction effect of Ma-bTMB for survival in patients receiving anti-PD-(L)1 therapy in Zhuo cohort, and found that high-Ma-bTMB (≥10) was significantly related with prolonged PFS (6.1 vs. 1.5 months, HR = 0.09, 95% CI 0.03–0.35; *p* < 0.001) and OS (17.6 vs. 6.6 months, HR = 0.23, 95% CI 0.07–0.78; *p* = 0.019) in patients with anti-PD-(L)1 therapy than low-Ma-bTMB (<10; Figure 2D). In the Wang cohort as the validation cohort, high-Ma-bTMB (≥10) was also significantly associated with improved PFS (3.3 vs. 2.0 months, HR = 0.45, 95% CI 0.22–0.94; *p* = 0.033) and OS (14.4 vs. 3.6 months, HR = 0.19, 95% CI 0.08–0.43; *p* < 0.001) compared with low-Ma-bTMB (Figure 2E).

### 3.4. Ma-bTMB as a Predictive Biomarker for Patients Receiving Anti-PD-(L)1 Therapy Rather Than Chemotherapy

**Figure 3:**
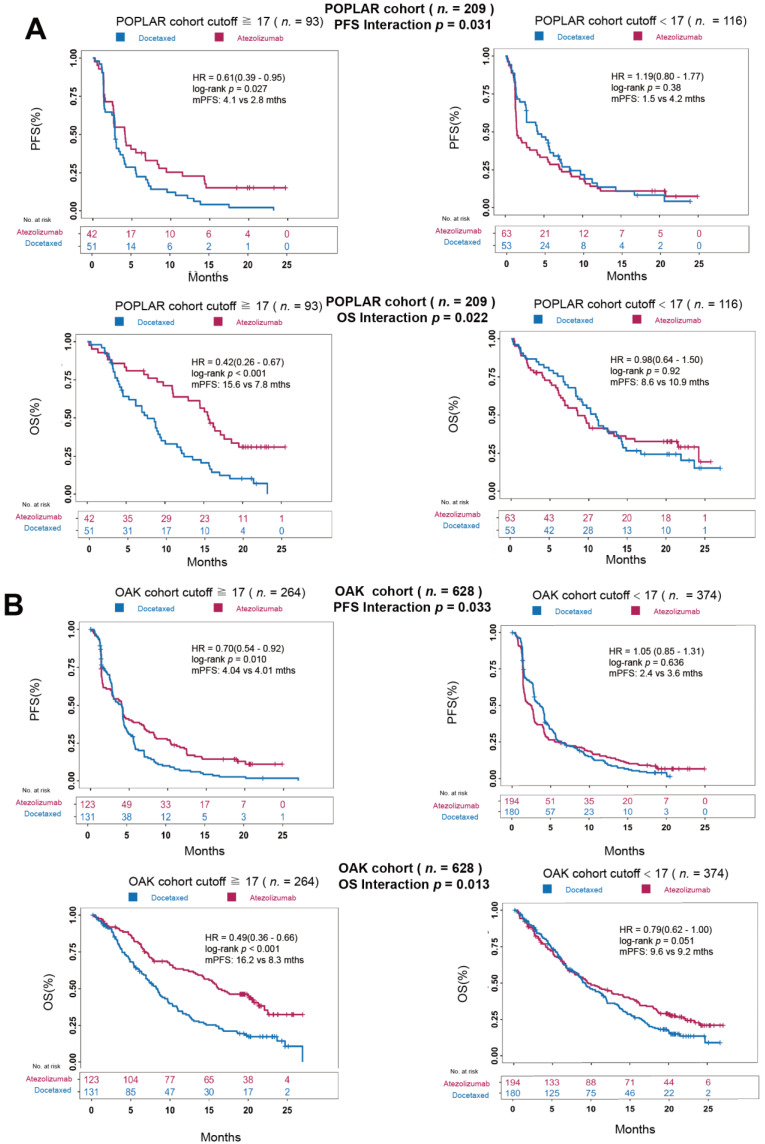
Further analysis of Ma-bTMB in POPLAR and OAK cohorts. (**A**) K–M curves of PFS and OS between the Ma-bTMB-H (**left** panel) and Ma-bTMB-L (**right** panel) groups in POPLAR cohort. (**B**) K–M curves of PFS and OS between the Ma-bTMB-H (**left** panel) and Ma-bTMB-L (**right** panel) groups in OAK cohort.

In the POPLAR cohort, the Ma-bTMB cutoff score of 17 was selected, with HR and *p*-value reaching a minimum in PFS prediction compared with other cutoffs (Figure S4). In patients with high-Ma-bTMB, atezolizumab was significantly associated with improved PFS and OS (PFS: 4.1 vs. 2.8 months, HR = 0.61, 95% CI 0.39–0.95, *p* = 0.027; OS: 15.6 vs. 7.8 months, HR = 0.42, 95% CI 0.26–0.67, *p* < 0.001, Figure 3A) over docetaxel. However, no difference was found in those with low-Ma-bTMB between two treatments (PFS: 1.5 vs. 4.1 months, HR = 1.19, 95% CI 0.80–1.77, *p* = 0.38; OS: 8.6 vs. 10.9 months, HR = 0.98, 95% CI 0.64–1.50, *p* = 0.92; Figure 3A). The interaction *p*-values for ICIs vs. chemotherapy treatment were significantly positive for PFS (*p* = 0.031) and OS (*p* = 0.022), as shown in Figure 3A. In the OAK cohort, similar results were observed. In the high-Ma-bTMB (≥17) group, patients receiving atezolizumab displayed a significant longer PFS (4.04 vs. 4.01 months, HR = 0.70, 95% CI 0.54–0.92, *p* = 0.010, Figure 3B) and longer OS (16.2 vs. 8.3 months, HR = 0.49, 95% CI 0.36–0.66; *p* < 0.001, Figure 3B) compared to those receiving docetaxel. However, no difference was found in the low-Ma-bTMB (<17) group (PFS: 2.4 vs. 3.6 months, HR = 1.05, 95% CI 0.85–1.31, *p* = 0.636; OS: 9.6 vs. 9.2 months, HR = 0.79, 95% CI 0.62–1.00, *p* = 0.051). The interaction *p*-values between Ma-bTMB and different treatment (atezolizumab vs. docetaxel) were significant in both PFS (Interaction Pfor PFS, 0.033) and OS (Interaction *p* = 0.013). Taken together, these results supported the notion that Ma-bTMB was proficient in identifying patients who may receive more benefit from ICIs therapy rather than chemotherapy.

### 3.5. A Comparison between Ma-bTMB and PD-L1 Expression

**Figure 4:**
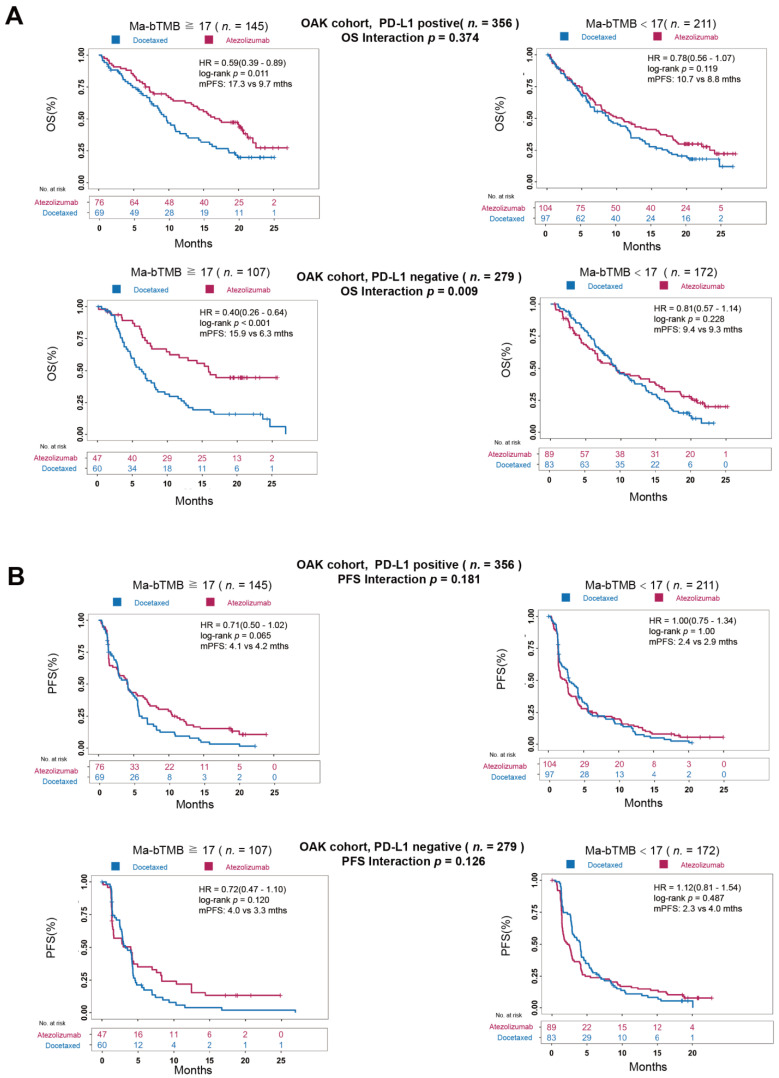
Association between Ma-bTMB and PD-L1 expression in OAK cohort. (**A**) K–M curves of PFS between the Ma-bTMB-H (**left** panel) and Ma-bTMB-L (**right** panel) groups in POPLAR cohort in patients with PD-L1 positive (**upper** panel) and PD-L1 negative expression (**bottom** panel) by atezolizumab and docetaxel treatment. (**B**) K–M curves of OS between the Ma-bTMB-H (**left** panel) and Ma-bTMB-L (**right** panel) groups in POPLAR cohort in patients with PD-L1 positive (**upper** panel) and PD-L1 negative expression (**bottom** panel) by atezolizumab and docetaxel treatment.

PD-L1 expression has been deemed a useful predictive biomarker before ICIs therapy in clinical practice [4]. We further explored the association of Ma-bTMB and PD-L1 expression in predicting survival benefit between atezolizumab and docetaxel in OAK cohort. In patients with PD-L1 positive expression/high-Ma-bTMB, atezolizumab was significantly associated with improved OS (17.3 vs. 9.7 months, HR = 0.59, 95% CI 0.39–0.89; *p* = 0.011, Figure 4A); however, no significant difference was found in those with PD-L1 positive expression/low-Ma-bTMB between two treatments (10.7 vs. 8.8 months, HR = 0.78, 95% CI 0.56–1.07; *p* = 0.119, Figure 4A). Notably, in patients with PD-L1 negative expression/high-Ma-bTMB, atezolizumab was still significantly associated with prolonged OS (15.9 vs. 6.3 months, HR = 0.40, 95% CI 0.26–0.64; *p* < 0.001, Figure 4A), and the interaction *p*-value for OS between Ma-bTMB and treatments was significant in PD-L1 negative expression group (interaction *P* for OS, 0.009). For PD-L1 negative expression group, there was similar trending for PFS benefit in patients receiving atezolizumab though without significant difference (PD-L1 negative expression/high-Ma-bTMB group: 4.0 vs. 3.3 months, HR = 0.72, 95% CI 0.47–1.10; *p* = 0.120; interaction *P* for PFS, 0.126; Figure 4B). Overall, our results indicated that high-Ma-bTMB could effectively identify potential ICIs beneficiaries, especially those with PD-L1 negative expression. 

### 3.6. A Comparison among Ma-bTMB, bTMB, and LAF-bTMB

**Figure 5:**
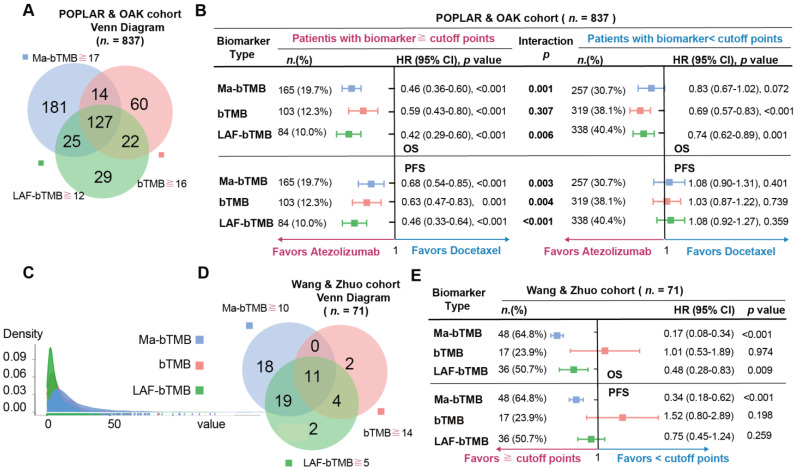
Comparison among bTMB, Ma-bTMB, and LAF-bTMB. (**A**) The Venn diagram showing the overlap of Ma-bTMB-H, bTMB-H, and LAF-bTMB-H in OAK & POPLAR cohort. (**B**) The forest plots for HRs, *p*-values, and interaction *p*-values of OS and PFS with corresponding cutoff points among Ma-bTMB, bTMB, and LAF-bTMB algorithms in OAK & POPLAR cohort. (**C**) The value distributions of Ma-bTMB, bTMB, and LAF-bTMB algorithms in OAK and POPLAR cohorts. (**D**) The Venn diagram showing the overlap of Ma-bTMB-H, bTMB-H, and LAF-bTMB-H in Wang & Zhuo cohort. (**E**) The forest plots for HRs, *p*-values, and interaction *p*-values of OS and PFS with corresponding cutoff points among Ma-bTMB, bTMB, and LAF-bTMB algorithms in Wang & Zhuo cohort.

We previously developed low allele frequency (LAF)-bTMB as a predictive biomarker in patients with advanced NSCLC receiving anti-PD-(L)1 [13]. We then compared the three biomarkers (Ma-bTMB, bTMB, and LAF-bTMB) in Wang & Zhuo and OAK & POPLAR cohorts. Firstly, we found that high-Ma-bTMB had more gentle trends covering more patients (Figure 5A). In the Wang & Zhuo cohort, high-Ma-bTMB not only could predict OS (HR = 0.17, 95% CI 0.08–0.34; *p* < 0.001) and PFS (HR = 0.34, 95% CI 0.18–0.62; *p* < 0.001) benefit from ICIs, as shown in Figure 5B, but also could identify more benefit populations compared with LAF-bTMB (64.8% vs. 50.7%, *p* = 0.126). However, bTMB could not predict OS (*p* = 0.974) nor PFS (*p* = 0.198) in the Wang & Zhuo cohort. In the OAK & POPLAR cohort, patients receiving atezolizumab were associated with significantly longer OS and PFS than patients receiving docetaxel among high-Ma-bTMB, high-bTMB, high-LAF-bTMB groups while the significant interaction *p*-values were only observed in Ma-bTMB (Interaction P for OS, 0.001; Interaction P for PFS, 0.003) and LAF-bTMB (Interaction P for PFS, 0.006; Interaction P for PFS, <0.001) biomarkers (Figure 5B,C). Though high-LAF-bTMB showed a lower HR than high-Ma-bTMB between atezolizumab and docetaxel, Ma-bTMB expanded the identification of potential ICI-benefited patients than LAF-bTMB (19.7% vs. 10.0%, *p* < 0.001; Figure 5D,E). In all, Ma-bTMB could steadily indicate both PFS and OS beneficiaries compared to bTMB, identifying more potential patients benefiting from ICIs than LAF-bTMB.

## 4. Discussion

In this study, we discovered that high-Ma-bTMB could predict the PFS and OS benefit of anti-PD-(L)1 therapy while serving as an effective predictive biomarker for selecting patients who could benefit from atezolizumab rather than chemotherapy. Patients with high-Ma-bTMB showed approximately 11 months longer OS in Zhuo (17.6 vs. 6.6 months) and Wang (14.4 vs. 3.6 months) cohorts after receiving ICIs, as well as about 8 months improved OS in POPLAR (15.6 vs. 7.8 months) and OAK (16.2 vs. 8.3 months) cohorts after receiving ICIs compared to chemotherapy. Even in patients with PD-L1 negative expression, those with high-Ma-bTMB receiving atezolizumab still had longer OS than chemotherapy. Our study showed that Ma-bTMB could steadily select ICIs beneficial populations in advanced NSCLC.

The establishment of Ma-bTMB is needed. With the revelation of subsequent clinical trials with long follow-ups, such as Keynote-001 and CheckMate-067, etc., it becomes clearer that ICIs therapy provides long-term benefits over conventional treatments, and identifying OS benefit is of great importance [16,17,18]. Although bTMB, whether tested by whole-exome sequencing or targeted gene panels, is deemed as a non-invasive biomarker candidate predicting the clinical benefit of ICIs therapy [7,8,19], recent evidence showed that bTMB failed to stably predict the survival benefit of ICIs therapy, particularly OS [9,10,13,20].

The establishment of Ma-bTMB is reasonable. Distinct from tTMB, the analysis of bTMB had been found to be impacted by several factors in the blood, including ctDNA, AF, MSAF grouping, ITH, etc. [13,20,21,22,23,24]. Considering that a patient with advanced NSCLC is characterized by high tumor burden and ITH, the relationship among bTMB, MSAF, and ITH has been analyzed. Consistent with previous studies [7,13,22], MSAF displayed a positive correlation with bTMB while negatively correlating with the survival of patients with advanced NSCLC receiving ICIs. Moreover, we found that MSAF was positively correlated with ITH. Hence, MSAF was hypothesized as the core confounding factor of bTMB in the prediction ability of ICIs therapy. In addition, previous evidence also indicated that MSAF alone was not an effective predictive biomarker of ICIs response [13]; hence, Ma-bTMB was established.

Genetically distinct cellular populations, resulting from clonal expansions, spatial segregation, and incomplete selective sweeps, were manifested as ITH [25]. Moreover, nearly all tumors display subclonal expansions, even at limited read depth [26]. Schmitt et al. evaluated the clonality of actionable driver mutations, reasoning that targeting subclonal mutations will likely result in treatment failure [27]. Considering the different numbers of subclones and subclone mutations, which were manifested as ITH, may also affect our evaluation of the subsequent treatment outcome; we analyzed their difference in different levels of Ma-bTMB. In our study, results showed that Ma-bTMB could decrease the effect of ITH on the stratification of beneficiaries and non-beneficiaries compared to bTMB. Different from the high-bTMB group presenting higher levels of subclonal mutations over the low-bTMB group, there were no differences between the high-Ma-bTMB and low-Ma-bTMB groups. In addition, this result is consistent with the evidence of the negative impact of ITH on PD-(L)1 inhibition therapy [28,29,30]. In other words, Ma-bTMB could decrease the effect of ITH on the stratification of beneficiaries and non-beneficiaries compared to bTMB, making it useful for predicting ICI-related benefits.

Although PD-L1 expression, tested via immunohistochemistry assays, is currently considered the most widely accepted biomarker guiding the selection of patients to receive anti-PD-L1/PD-1 treatment [31], patients with low PD-L1 expression could also benefit from anti-PD-L1/PD-1 treatment [4,32]. Indeed, the interaction *P* for OS between Ma-bTMB and treatment subgroups was significant in patients with PD-L1 negative expression and not significant in patients with PD-L1 positive expression. Patients with PD-L1 positive expression and high-Ma-bTMB showed the longest OS benefit (17.3 months) among all, and those with high-Ma-bTMB had 9 months longer OS after ICI therapy over chemotherapy (15.9 vs. 6.3 months) in patients with PD-L1 negative expression. Intriguingly, regardless of PD-L1 expression, patients with low-Ma-bTMB receiving ICIs therapy showed neither PFS nor OS benefit compared to chemotherapy. In this scenario, a combination biomarker of PD-L1 expression and Ma-bTMB analysis could guide the treatment selection between ICIs and chemotherapy.

Previously, we developed an adjusted bTMB algorithm, LAF-bTMB, by removing high AF mutations to predict the clinical benefit of PD-(L)1 inhibitors in patients with advanced NSCLC [13]. In this study, we compared Ma-bTMB, bTMB, and LAF-bTMB in POPLAR&OAK and Zhuo & Wang cohorts. Unfortunately, bTMB could not effectively identify the OS benefit of ICIs therapy in either cohort. Compared to LAF-bTMB, Ma-bTMB could identify more potential OS and PFS beneficiaries in both cohorts. The value distributions of three biomarkers showed that LAF-bTMB had the highest and narrowest “slim” curve while Ma-bTMB had the lowest and widest “flat” curve, indicating that Ma-bTMB could better disperse the population and reduce the accumulation of patients according to the characteristic of bTMB. Given that patients with higher Ma-bTMB/bTMB/LAF-bTMB have better ICIs response, the “flat” curve may decrease the selection bias caused by the definition of the cutoff value. Hence, Ma-bTMB could steadily identify more beneficiaries to minimize potential patients who miss the chance of ICIs benefits.

There are several limitations to this study. First, the algorithm was developed retrospectively, and the sample size of the Wang and Zhuo cohorts was relatively small, which could bias the results. In addition, the detection platforms and methodologies differed in POPLAR&OAK and Wang & Zhuo cohorts. A larger prospective study is warranted for further validation. Nevertheless, the stable prediction ability of Ma-bTMB between these two platforms (1021-gene-panel and F1CDx NGS assay) and four cohorts (Zhuo, Wang, POPLAR, and OAK) indicated its feasibility, to some extent, as a predictive biomarker of ICI benefit in patients with advanced NSCLC.

## 5. Conclusions

In summary, although a larger prospective study is necessary for further verification, we developed and validated a convenient and readily available novel algorithm, Ma-bTMB, that can predict clinical benefits to guide ICI therapy in patients with advanced NSCLC. 

## Data Availability

All datasets used and analyzed in this study are available from the corresponding authors for reasonable request.

## Supplementary Materials

The following supporting information can be downloaded at: https://www.mdpi.com/article/10.3390/cancers14225649/s1, Figure S1:The forest plots for HRs and *p*-values of OS and PFS comparing bTMB-H and bTMB-L subgroups with corresponding cutoff points in Zhuo cohort and Wang cohort; Figure S2: The correlation between MSAF and OS/PFS in Wang & Zhuo cohort; Figure S3: The distribution of subclones between high MSAF and low MSAF changed with MSAF cutoff points in GCGD cohort. Figure S4: The forest plots for HRs and *p* values of PFS between atezolizumab and docetaxel comparing Ma-bTMB-H and Ma-bTMB-L subgroups with corresponding cutoff points in POPLAR cohort. Figure S5: The forest plots for HRs and *p*-values of OS comparing LAF-bTMB-H and LAF-bTMB-L changed with corresponding cutoff points in Wang & Zhuo cohort. Table S1: Basic characteristic of patients in Wang cohort; Table S2: Basic characteristic of patients in Zhuo cohort; Table S3: Clinical data and somatic mutations of Wang & Zhuo cohort; Table S4: Clinical data and somatic mutations of OAK & POPLAR cohort.

## Abbreviations

MSAF	maximum somatic allele frequency;
bTMB	blood-based tumor mutational burden
tTMB	tissue-based tumor mutational burden
AF	allele frequency
OS	overall survival
PFS	progression-free survival
HR	hazard ratio
NSCLC	non-small cell lung cancer
ICIs	immune checkpoint inhibitors
GCGD	Geneplus Cancer Genome Database
Ma-bTMB	MSAF-adjusted bTMB
LAF-bTMB	low allele frequency-bTMB
ITH	intratumor heterogeneity
PD-1	programmed death 1
PD-L1	programmed death ligand 1
ctDNA	circulating tumor DNA

## References

[1] Rittmeyer A., Barlesi F., Waterkamp D., Park K., Ciardiello F., von Pawel J., Gadgeel S.M., Hida T., Kowalski D.M., Dols M.C. (2017). Atezolizumab versus docetaxel in patients with previously treated non-small-cell lung cancer (OAK): A phase 3, open-label, multicentre randomised controlled trial. Lancet.

[2] Borghaei H., Gettinger S., Vokes E.E., Chow L.Q.M., Burgio M.A., de Castro Carpeno J., Pluzanski A., Arrieta O., Frontera O.A., Chiari R. (2021). Five-Year Outcomes From the Randomized, Phase III Trials CheckMate 017 and 057: Nivolumab Versus Docetaxel in Previously Treated Non-Small-Cell Lung Cancer. J. Clin. Oncol..

[3] Fehrenbacher L., Spira A., Ballinger M., Kowanetz M., Vansteenkiste J., Mazieres J., Park K., Smith D., Artal-Cortes A., Lewanski C. (2016). Atezolizumab versus docetaxel for patients with previously treated non-small-cell lung cancer (POPLAR): A multicentre, open-label, phase 2 randomised controlled trial. Lancet.

[4] Herbst R.S., Giaccone G., de Marinis F., Reinmuth N., Vergnenegre A., Barrios C.H., Morise M., Felip E., Andric Z., Geater S. (2020). Atezolizumab for First-Line Treatment of PD-L1-Selected Patients with NSCLC. N. Engl. J. Med..

[5] Marabelle A., Fakih M., Lopez J., Shah M., Shapira-Frommer R., Nakagawa K., Chung H.C., Kindler H.L., Lopez-Martin J.A., Miller W.H. (2020). Association of tumour mutational burden with outcomes in patients with advanced solid tumours treated with pembrolizumab: Prospective biomarker analysis of the multicohort, open-label, phase 2 KEYNOTE-158 study. Lancet Oncol..

[6] Lim C., Tsao M.S., Le L.W., Shepherd F.A., Feld R., Burkes R.L., Liu G., Kamel-Reid S., Hwang D., Tanguay J. (2015). Biomarker testing and time to treatment decision in patients with advanced nonsmall-cell lung cancer. Ann. Oncol..

[7] Gandara D.R., Paul S.M., Kowanetz M., Schleifman E., Zou W., Li Y., Rittmeyer A., Fehrenbacher L., Otto G., Malboeuf C. (2018). Blood-based tumor mutational burden as a predictor of clinical benefit in non-small-cell lung cancer patients treated with atezolizumab. Nat. Med..

[8] Si H., Kuziora M., Quinn K.J., Helman E., Ye J., Liu F., Scheuring U., Peters S., Rizvi N.A., Brohawn P.Z. (2021). A Blood-based Assay for Assessment of Tumor Mutational Burden in First-line Metastatic NSCLC Treatment: Results from the MYSTIC Study. Clin. Cancer Res..

[9] Kim E.S., Velcheti V., Mekhail T., Yun C., Shagan S.M., Hu S., Chae Y.K., Leal T.A., Dowell J.E., Tsai M.L. (2022). Blood-based tumor mutational burden as a biomarker for atezolizumab in non-small cell lung cancer: The phase 2 B-F1RST trial. Nat. Med..

[10] Nie W., Wang Z.J., Zhang K., Li B., Cai Y.R., Wen F.C., Zhang D., Bai Y.Z., Zhang X.Y., Wang S.Y. (2022). ctDNA-adjusted bTMB as a predictive biomarker for patients with NSCLC treated with PD-(L)1 inhibitors. BMC Med..

[11] Ulz P., Thallinger G.G., Auer M., Graf R., Kashofer K., Jahn S.W., Abete L., Pristauz G., Petru E., Geigl J.B. (2016). Inferring expressed genes by whole-genome sequencing of plasma DNA. Nat. Genet..

[12] De Mattos-Arruda L., Weigelt B., Cortes J., Won H.H., Ng C.K.Y., Nuciforo P., Bidard F.C., Aura C., Saura C., Peg V. (2018). Capturing intra-tumor genetic heterogeneity by de novo mutation profiling of circulating cell-free tumor DNA: A proof-of-principle. Ann. Oncol..

[13] Wang Z., Duan J., Wang G., Zhao J., Xu J., Han J., Zhao Z., Zhao J., Zhu B., Zhuo M. (2020). Allele Frequency-Adjusted Blood-Based Tumor Mutational Burden as a Predictor of Overall Survival for Patients with NSCLC Treated with PD-(L)1 Inhibitors. J. Thorac. Oncol..

[14] Jamal-Hanjani M., Quezada S.A., Larkin J., Swanton C. (2015). Translational implications of tumor heterogeneity. Clin. Cancer Res..

[15] Fang W., Jin H., Zhou H., Hong S., Ma Y., Zhang Y., Su X., Chen L., Yang Y., Xu S. (2021). Intratumoral heterogeneity as a predictive biomarker in anti-PD-(L)1 therapies for non-small cell lung cancer. Mol. Cancer.

[16] Garon E.B., Hellmann M.D., Rizvi N.A., Carcereny E., Leighl N.B., Ahn M.J., Eder J.P., Balmanoukian A.S., Aggarwal C., Horn L. (2019). Five-Year Overall Survival for Patients with Advanced Non–Small-Cell Lung Cancer Treated with Pembrolizumab: Results From the Phase I KEYNOTE-001 Study. J. Clin. Oncol..

[17] Gauci M.L., Lanoy E.A.-O., Champiat S., Caramella C., Ammari S.A.-O., Aspeslagh S., Varga A., Baldini C., Bahleda R., Gazzah A. (2019). Long-Term Survival in Patients Responding to Anti-PD-1/PD-L1 Therapy and Disease Outcome upon Treatment Discontinuation. Clin. Cancer Res..

[18] Wolchok J.A.-O., Chiarion-Sileni V.A.-O., Gonzalez R., Grob J.A.-O.X., Rutkowski P.A.-O., Lao C.D., Cowey C.L., Schadendorf D.A.-O., Wagstaff J.A.-O., Dummer R.A.-O. (2022). Long-Term Outcomes with Nivolumab Plus Ipilimumab or Nivolumab Alone Versus Ipilimumab in Patients with Advanced Melanoma. J. Clin. Oncol..

[19] Wang Z., Duan J., Cai S., Han M., Dong H., Zhao J., Zhu B., Wang S., Zhuo M., Sun J. (2019). Assessment of Blood Tumor Mutational Burden as a Potential Biomarker for Immunotherapy in Patients with Non-Small Cell Lung Cancer with Use of a Next-Generation Sequencing Cancer Gene Panel. JAMA Oncol..

[20] Owada-Ozaki Y., Muto S., Takagi H., Inoue T., Watanabe Y., Fukuhara M., Yamaura T., Okabe N., Matsumura Y., Hasegawa T. (2018). Prognostic Impact of Tumor Mutation Burden in Patients with Completely Resected Non-Small Cell Lung Cancer: Brief Report. J. Thorac. Oncol..

[21] Fang W., Ma Y., Yin J.C., Zhou H., Wang F., Bao H., Wang A., Wu X., Hong S., Yang Y. (2020). Combinatorial assessment of ctDNA release and mutational burden predicts anti-PD(L)1 therapy outcome in nonsmall-cell lung cancer. Clin. Transl. Med..

[22] Chen Y.T., Seeruttun S.R., Wu X.Y., Wang Z.X. (2019). Maximum Somatic Allele Frequency in Combination with Blood-Based Tumor Mutational Burden to Predict the Efficacy of Atezolizumab in Advanced Non-small Cell Lung Cancer: A Pooled Analysis of the Randomized POPLAR and OAK Studies. Front. Oncol..

[23] Liu Z., Xie Z., Cai X., He J., Liang W. (2020). A Modified Algorithm Adjusting Both High and Minor Allele Frequency Mutation to Redefine Blood-Based Tumor Mutational Burden (bTMB) for Optimal Prediction of Clinical Benefits From Immune Checkpoint Inhibitor Therapy. J. Thorac. Oncol..

[24] Tang Y., Liu X., Ou Z., He Z., Zhu Q., Wang Y., Yang M., Ye J., Zhang H.H., Qiao G. (2019). Maximum allele frequency observed in plasma: A potential indicator of liquid biopsy sensitivity. Oncol. Lett..

[25] Nowell P.C. (1976). The clonal evolution of tumor cell po pulations. Science.

[26] Dentro S.C., Leshchiner I., Haase K., Tarabichi M., Wintersinger J., Deshwar A.G., Yu K., Rubanova Y., Macintyre G., Demeulemeester J. (2021). Characterizing genetic intra-tumor heterogeneity across 2658 human cancer genomes. Cell.

[27] Schmitt M.W., Loeb L.A., Salk J.J. (2016). The influence of subclonal resistance mutations on targeted cancer therapy. Nat. Rev. Clin. Oncol..

[28] Yu S., Murph M.M., Lu Y., Liu S., Hall H.S., Liu J., Stephens C., Fang X., Mills G.B. (2008). Lysophosphatidic acid receptors determine tumorigenicity and aggressiveness of ovarian cancer cells. J. Natl. Cancer Inst..

[29] Landau D.A., Carter S.L., Stojanov P., McKenna A., Stevenson K., Lawrence M.S., Sougnez C., Stewart C., Sivachenko A., Wang L. (2013). Evolution and impact of subclonal mutations in chronic lymphocytic leukemia. Cell.

[30] Wolf Y., Bartok O., Patkar S., Eli G.B., Cohen S., Litchfield K., Levy R., Jiménez-Sánchez A., Trabish S., Lee J.S. (2013). UVB-Induced Tumor Heterogeneity Diminishes Immune Response in Melanoma. Cell.

[31] Loperfido F., Laurenzi F., Gimigliano F., Pennestri F., Biasucci L.M., Vigna C., Santis F.D., Favuzzi A., Rossi E., Manzoli U. (1987). A comparison of the assessment of mitral valve area by continuous wave Doppler and by cross sectional echocardiography. Br. Heart J..

[32] Carbone D.P., Reck M., Paz-Ares L., Creelan B., Horn L., Steins M., Felip E., van den Heuvel M.M., Ciuleanu T.E., Badin F. (2019). First-Line Nivolumab in Stage IV or Recurrent Non-Small-Cell Lung Cancer. N. Engl. J. Med..

