# Supplementary material for: Maximum Somatic Allele Frequency-Adjusted Blood-Based Tumor Mutational Burden Predicts the Efficacy of Immune Checkpoint Inhibitors in Advanced Non-Small Cell Lung Cancer
Source: Cancers (Basel). 2022 Nov 17;14(22):5649. doi: 10.3390/cancers14225649 (PMC9688065; doi:10.3390/cancers14225649)
Supplement: Supplementary file 1 [file cancers-14-05649-s001.zip › Supplementary Files(FigureS1-S5+TableS1-S2).pdf]

Supplementary Files

Figure S1:The forest plots for HRs and P- values of OS and PFS comparing bTMB-H and bTMB-L subgroups with corresponding cutoff points in Zhuo cohort and Wang cohort.

Figure S2:The correlation between MSAF and OS/PFS in Wang & Zhuo cohort.

Figure S3:The distribution of subclones between high MSAF and low MSAF changed with MSAF cutoff points in GCGD cohort.

Figure S4:The forest plots for HRs and P values of PFS between atezolizumab and docetaxel comparing Ma-bTMB-H and Ma-bTMB-L subgroups with corresponding cutoff points in POPLAR cohort.

Figure S5:The forest plots for HRs and P- values of OS comparing LAF-bTMB-H and LAF-bTMB-L changed with corresponding cutoff points in Wang & Zhuo cohort.

Figure S1

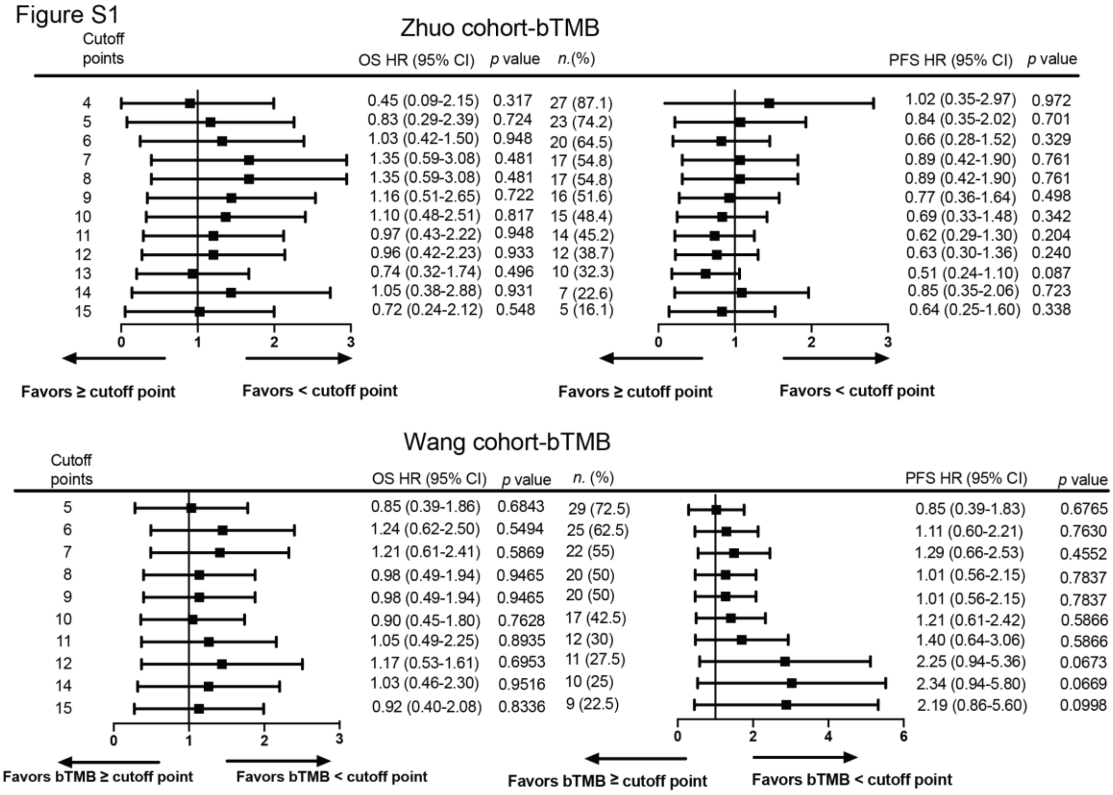

Figure S2

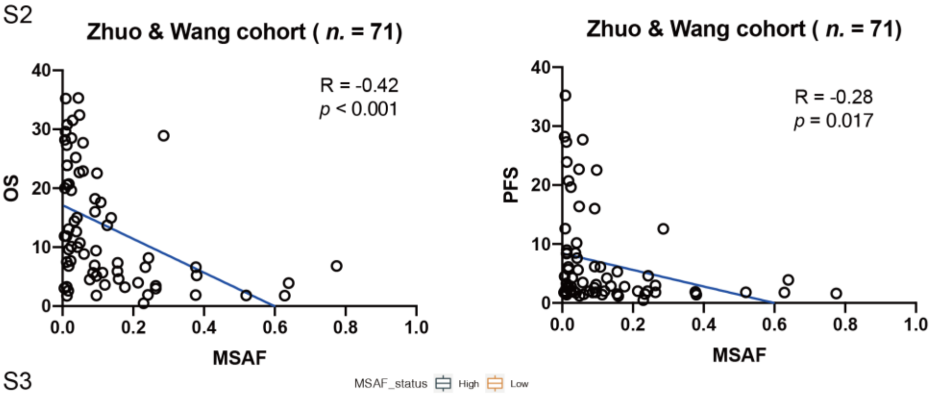

Figure S3

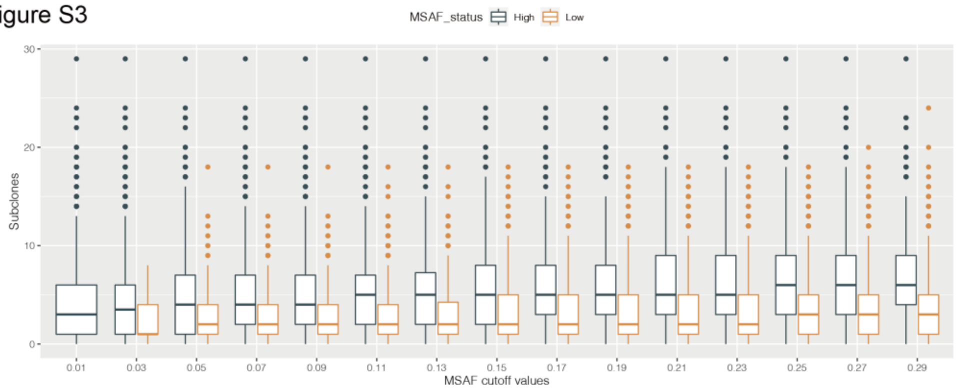

Figure S4

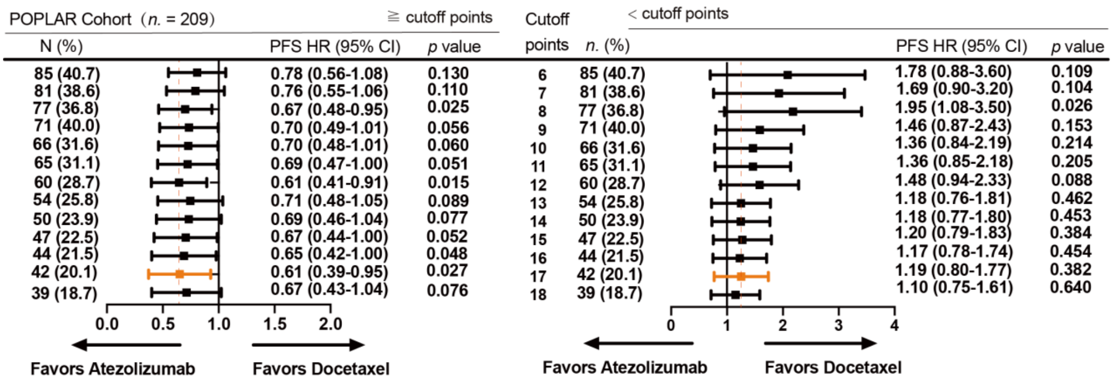

Figure S5

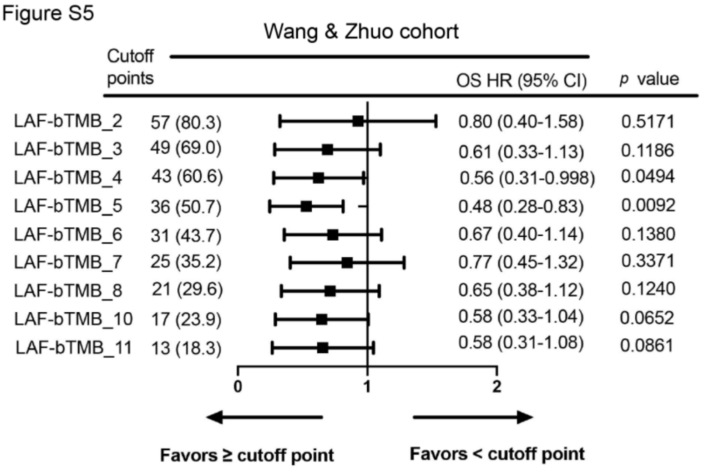

Table S1: Basic characteristic of patients in Wang cohort.

Characteristics	Total (n = 40)
Age (years), median (range)	58 (39-69)
Gender (% female)	10 (25)
ECOG PS (%)	
0-1	35 (87.5)
≥ 2	4 (10)
Unknown	1 (2.5)
Smoking status (%)	
Never	13 (32.5)
Former/current	26 (65)
Unknown	1 (2.5)
Histology (%)	
Non-squamous	28 (67.5)
Squamous	12 (32.5)
Stage (%)	
III	7 (17.5)
IV	33 (82.5)
bTMB (muts/Mb), median (range)	7 (1-38)

ECOG, Eastern Cooperative Oncology Group; PS, performance status; bTMB, blood tumor mutational burden.

Table S2: Basic characteristic of patients in Zhuo cohort.

Characteristics	Total (n = 31)
Age (years), median (range)	62 (47-77)
Gender (% female)	6 (19.3)
ECOG PS (%)	
0-1	30 (96.8)
≥ 2	1 (3.2)
Smoking status (%)	
Never	9 (29.1)
Former/current	22 (70.9)
Histology (%)	
Non-squamous	24 (77.5)
Squamous	7 (22.5)
Stage (%)	
III	5 (16.1)
IV	26 (83.9)
bTMB (muts/Mb), median (range)	8.64 (0.96-48.08)

ECOG, Eastern Cooperative Oncology Group; PS, performance status; bTMB, blood tumor mutational burden.
